# Multi-Scale simulation of electromagnetic wave excitation by positive corona discharge in SF_6_ gas

**DOI:** 10.1038/s41598-025-01904-4

**Published:** 2025-05-26

**Authors:** Feng Bin, Chuanfei Yao, Jixiang Feng, Fangwei Liang, Qiuqin Sun

**Affiliations:** 1 School of Physics and Electronic Science, Changsha University of Science and Technology, Changsha, 410114 China; 2 College of Electrical and Information Engineering, Hunan University, Changsha, 410082 China; 3 State Key Laboratory of Power Systems, Tsinghua University, Beijing, 100084 China

**Keywords:** Finite-difference time-domain (FDTD) method, Corona discharge, Fluid dynamics model, Multi-scale simulation, Electromagnetic (EM) wave, Electrical and electronic engineering, Energy science and technology

## Abstract

Corona discharge is a typical discharge in gas-insulated equipment; however, the correlation between microscopic discharge process and macroscopic electromagnetic (EM) wave signals excited by discharge remains unclear. Therefore, this study innovatively employs the space current pulse as a bridge to reveal their relationship through the multi-scale simulation. First, the needle-plate discharge process in SF_6_ gas is simulated based on a fluid dynamics model. Then, the effects of voltage, temperature, and the curvature of needle tip on the space current pulse are investigated. Lastly, the current pulses generated under varying conditions serve as excitation sources, and the finite-difference time-domain (FDTD) method is utilized to establish correlations between the corona discharge stages and discharge conditions and the amplitude-frequency characteristics of excited EM waves. The simulation results indicate that in the rising and falling stages of current pulse, the spectral energy is predominantly concentrated in the high frequency band (2.3–3.0 GHz) of the ultra-high-frequency (UHF) range, whereas the spectral energy constitutes the highest proportion within the mid-high frequency band (1.6–2.3 GHz) in the stabilization stage. As voltage, temperature, or the curvature of needle tip increases, there is a corresponding rise in the proportion of EM energy within both the low frequency band (0.2–0.9 GHz) and the mid-low frequency band (0.9–1.6 GHz), as well as in the mid-high frequency band; conversely, the proportion of energy within the high frequency band diminishes. The proposed multi-scale simulation method provides a novel way to obtain the characteristics of EM waves induced by partial discharge (PD) in gas.

## Introduction

Gas-insulated switchgear (GIS) is a class of electrical equipment with SF_6_ gas as an insulating material^1^. They have the advantages of superior insulation performance, enhanced safety, and strong environmental adaptability, making them indispensable components of power systems^2^. In the manufacturing, transportation, installation, and routine operation of GIS, internal defects inevitably occur, leading to partial discharge (PD)^3,4^. By detecting the electromagnetic (EM) wave signals generated by PD, the online monitoring of GIS can be carried out, enabling the timely discovery of potential insulation faults^5–8^. At present, the characteristics of EM waves generated under various PD conditions are mainly obtained through experimental methods. However, PD experiments are associated with several drawbacks, such as high costs, limited controllability, long duration, and significant safety risks^9,10^. In contrast, the utilization of simulation technology can attain the EM wave signals from different PD conditions by merely adjusting simulation parameters^11–14^. This eliminates the need for physical experiments. Therefore, multi-scale simulations which combine the microscopic discharge process with the macroscopic EM wave phenomena offer an effective alternative to traditional experimental methods, and it provides substantial benefits, including cost reduction, simplified implementation, shorter response times, and improved safety.

The research on microscopic discharge processes in gases has achieved remarkable progress in recent years. For instance, Li et al. utilized numerical modeling to elucidate the dynamic behavior of electrons, ions, and neutral particles during air discharge processes^15^. Peng et al. analyzed the generation, acceleration, and ionization processes of electrons under high-voltage conditions, providing a theoretical foundation for the early warning and diagnosis of PD^16^. Espel et al. investigated numerical simulation methods for negative corona discharges in SF_6_ gas at standard atmospheric pressure, revealing the roles of positive ion bombardment and secondary electron emission in gas breakdown^17^. Morrow et al. employed the flux corrected transport (FCT) algorithm to study SF_6_ gas discharge characteristics under constant voltage, illuminating the influence of interactions between electrons, ions, and electric fields on the discharge process^18^. Liu et al. applied an axisymmetric hybrid model to simulate DC discharge in air at atmospheric pressure, predicting discharge V-C characteristics and current density distributions^19^. Wang et al. combined discharge spectroscopy with V-C characteristics to explore the microscopic particle reaction mechanisms in N_2_ gas discharges under various pressure and temperature conditions^20^.

Similarly, scholars have conducted extensive experimental investigations on the macroscopic EM wave characteristics excited by PD over the past few years. Hoshino et al. utilized a disc antenna to detect EM wave signals generated by insulation defect-induced discharges in GIS, observing their frequency components up to 8 GHz^21^. Xu et al. analyzed the frequency characteristics of transient EM wave excited by PD sources at diffident locations in enclosures and elucidated the spatiotemporal variations and attenuation laws of EM wave propagation^22^. Through high-voltage experiments, Wang et al. discovered that as the applied voltage increased, both the amplitude and spectral range of EM waves expanded significantly^23^. Javandel et al. conducted comparative studies on EM wave signals produced by positive and negative corona discharges in air, revealing that the former exhibited higher energy at higher frequencies whereas the inverse relationship held at lower frequencies^24^. Wu et al. experimentally ascertained that the average attenuation rate of EM wave signals induced by PD was 0.37 dB/m when propagating in a straight line within a 252 kV GIS^25^. Gharehpetian et al. explored the EM wave spectral characteristics at different discharge stages, and the experimental results indicated that they could serve as the key indicators for recognizing discharge types and localizing PD sources^26^.

**Fig. 1:**
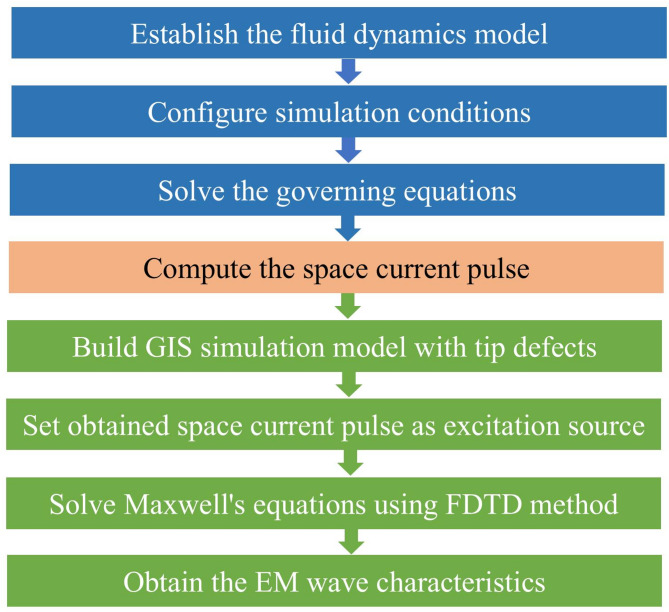
Flowchart of our proposed multi-scale simulation method.

Nevertheless, current research typically regards the microscopic gas discharge process and the EM waves induced by PD as two independent stages, failing to effectively establish a correlation between them. To tackle this issue, we adopt multi-scale simulation (i.e., a cross-scale simulation that bridges microscopic discharge process with macroscopic EM wave characteristics through space current pulse) to analyze the effects of voltage, temperature, and tip curvature on the characteristics of EM waves excited by positive corona discharge in SF_6_ gas. The flowchart is shown in Fig. 1.

## Simulation of corona discharge in SF_6_ gas

### Simulation model

**Fig. 2:**
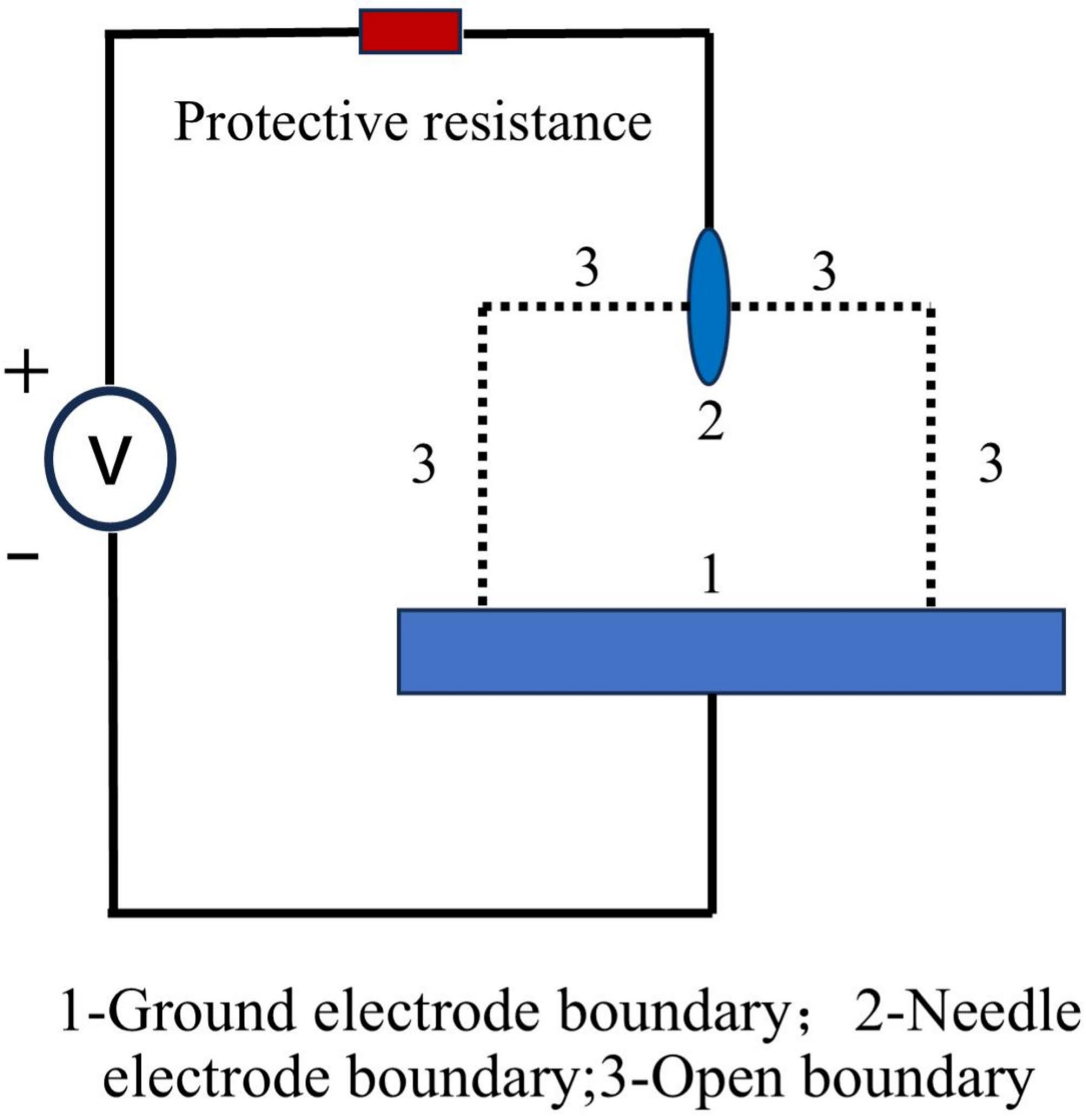
Simulation model of positive corona discharge.

**Fig. 3:**
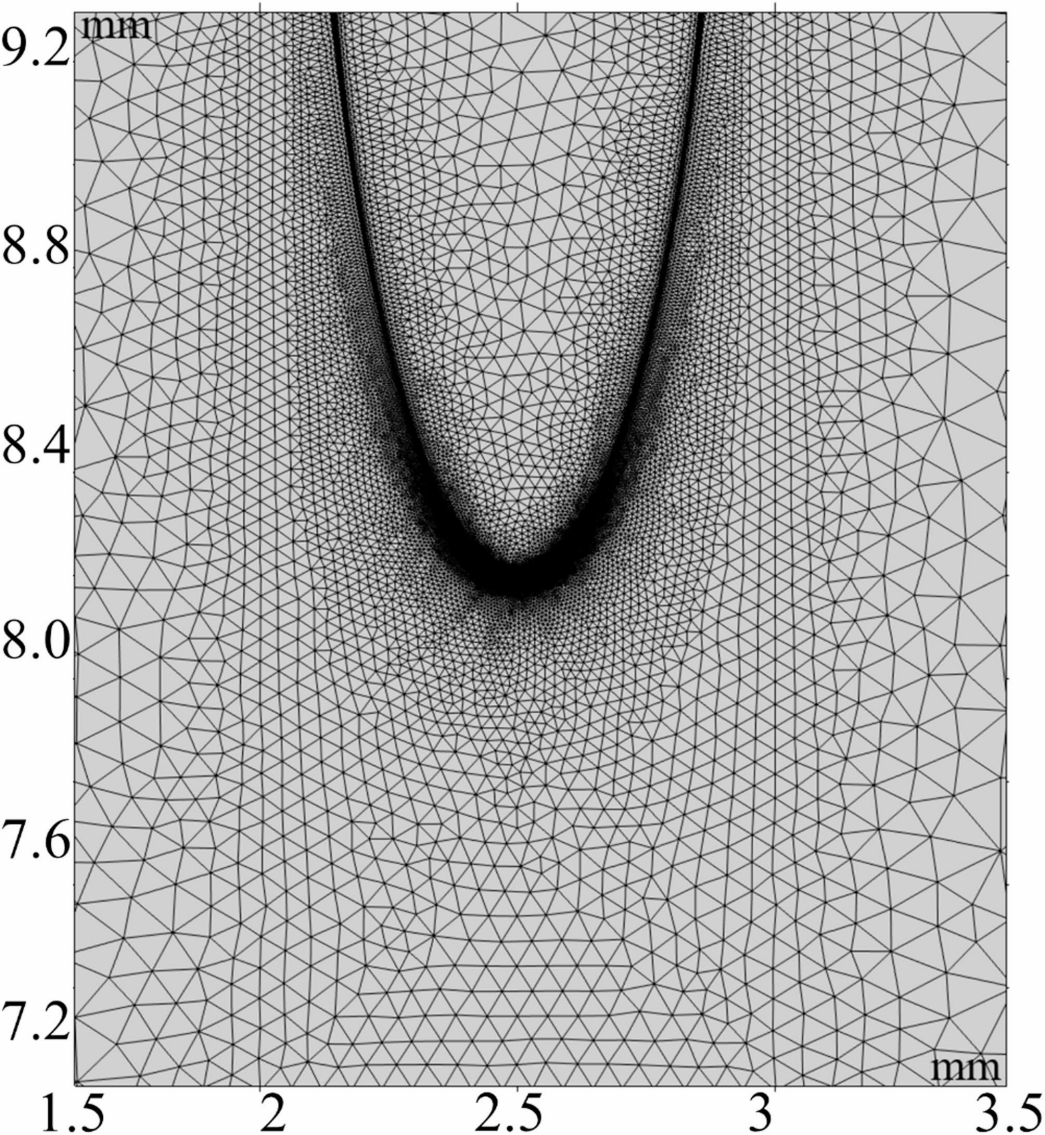
Mesh division.

To examine the positive corona discharge characteristics of SF_6_ gas, a 2D needle-plate discharge model, as illustrated in Fig. 2, is built using COMSOL. The plate electrode is grounded, the protection resistance is 1 kΩ, the insulating material is SF_6_ gas, and the computational domain is 5 mm wide and 10 mm long. The reference conditions in the simulation are defined as follows: voltage *V* and temperature *T* are set to 36 kV and 300 K, respectively, with the major axis of needle electrode *a* = 2 mm and the minor axis *b* = 0.4 mm. Since corona discharge primarily occurs near the tip electrodes, this region is continually refined with a free triangular mesh. The mesh division is presented in Fig. 3.

### Governing equations

Based on fluid dynamics, the numerical modeling of corona discharge consists of three continuity equations for electrons, positive ions, and negative ions, which are coupled with Poisson’s equation, to characterize the generation, dissipation, and transport processes of charged particles. The governing equations are given by^27^

1 $$\left\{ \begin{gathered} \frac{{\partial {N_p}}}{{\partial t}}+\nabla \cdot ( - {\mu _p}E{N_p} - D\nabla {N_p})={S_p} \\ \frac{{\partial {N_n}}}{{\partial t}}+\nabla \cdot ( - {\mu _n}E{N_n} - D\nabla {N_n})={S_n} \\ \frac{{\partial {N_e}}}{{\partial t}}+\nabla \cdot ( - {\mu _e}E{N_e} - D\nabla {N_e})={S_e} \\ {\nabla ^2}\varphi =\frac{{ - e({N_p} - {N_e} - {N_n})}}{{{\varepsilon _r}{\varepsilon _0}}} \\ \end{gathered} \right.$$

where *t* indicates time; *N*_*p*_, *N*_*n*_, and *N*_*e*_ represent the positive ion, negative ion, and electron number densities, respectively; *E* signifies the electric field strength; *µ*_p_, *µ*_n_, and *µ*_e_ are the material mobilities of positive ions, negative ions, and electrons, respectively; *D* denotes the material diffusion coefficient; *S*_*p*_, *S*_*n*_, and *S*_*e*_ stand for the reaction source terms of positive ions, negative ions, and electrons, respectively; *e* is the charge of an electron; *φ* symbolizes the electric potential; *ε*_*r*_ refers to the relative dielectric constant; *ε*_*0*_ means the vacuum dielectric constant.

The relationship between the reaction source term and the transport parameters of particles is given by^28^

2 $$\left\{ \begin{gathered} {S_e}=a{N_e}|{\mu _e}E| - \eta {N_e}|{\mu _e}E| - \beta {N_e}{N_p} \\ {S_p}=a{N_e}|{\mu _e}E| - \beta {N_e}{n_p} - \beta {N_n}{N_p} \\ {S_n}=\eta {N_e}|{\mu _e}E| - \beta {N_n}{N_p} \\ \end{gathered} \right.$$

where *α*, *η*, and *β* are the ionization coefficient, attachment coefficient, and recombination coefficient, respectively. The reaction parameters under different conditions, such as temperatures and gas types, can be computed using the Bolsig + software^29^.

### Chemical parameters

To simplify the reaction process, only four categories of particles30, i.e., SF_6_, SF_6_^+^, SF_6_^-^ and electrons, are considered. The four chemical reactions involved are detailed in Table 1, and the reaction parameters in the governing equations can be found in Table 2, where *P* stands for gas pressure and *N* represents the total number density of SF_6_ gas. At the initial time, a uniform distribution of electrons is introduced within the gap, with an initial electron number density of 1 × 10^14^ m^− 3^. The initial number density ratios of charged particles are set as SF_6_^+^: SF_6_^−^: e = 2:1:1, which satisfies the electric neutrality conservation of charged particles. The needle-plate discharge model involves three external boundaries^32^: the ground electrode boundary, the needle electrode boundary, and the open boundary. The boundary conditions are specified in Table 3.

**Table 1:** Chemical reactions^30^.

Reaction equation	Reaction type
$e+S{F_6} \to 2e+S{F_6}^{+}$	Lonization
$e+S{F_6} \to S{F_6}^{ - }$	Attachment
$e+S{F_6}^{+} \to S{F_6}$	Neutralization
$S{F_6}^{ - }+S{F_6}^{+} \to 2S{F_6}$	Neutralization

**Table 2:** Reaction parameters^31^.

Parameters	Value	Unit
*α*	$3.4473 \times {10^{34}} \times {(E/N)^{2.985}} \times N$	m^− 1^
*η*	$7.0 \times {10^{ - 21}} \times {e^{ - 2.25 \times {{10}^{118}} \times E/N}} \times N$	m^− 1^
*β*	$2.28 \times {10^{ - 11}} \times {P^{ - 0.659}}$	m^3^∙s^− 1^

**Table 3:** Boundary conditions^32^.

Governing equations	Ground electrode boundary	Needle electrode boundary	Open boundary
Electron transport equation	Outflow flux$- \vec {n} \cdot D\nabla {N_e}=0$	Flux of secondary electrons	$$\begin{array}{*{20}{c}} { - \vec {n} \cdot D\nabla {N_e}=0;\vec {n} \cdot ( - {\mu _e}\vec {E}) \geqslant 0} \\ {{N_e}=0;\vec {n} \cdot ( - {\mu _e}\vec {E})<0} \end{array}$$
Positive ion transport equation	Zero density${N_p}=0$	Outflow flux$- \vec {n} \cdot D\nabla {N_p}=0$	$$\begin{array}{*{20}{c}} { - \vec {n} \cdot D\nabla {N_p}=0;\vec {n} \cdot ( - {\mu _p}\vec {E}) \geqslant 0} \\ {{N_p}=0;\vec {n} \cdot ( - {\mu _p}\vec {E})<0} \end{array}$$
Negative ion transport equation	Outflow flux$- \vec {n} \cdot D\nabla {N_n}=0$	Zero density${N_n}=0$	$$\begin{array}{*{20}{c}} { - \vec {n} \cdot D\nabla {N_n}=0;\vec {n} \cdot ( - {\mu _n}\vec {E}) \geqslant 0} \\ {{N_n}=0;\vec {n} \cdot ( - {\mu _n}\vec {E})<0} \end{array}$$
Poisson equation	Ground potential	Positive DC potential	Zero charge$\vec {n} \cdot ({\varepsilon _r}{\varepsilon _0}\vec {E})=0$

### Results and discussion

The charged particles motion, induced by an electric field, alters to the spatial charge distribution, subsequently giving rise to a space current pulse, whose calculation formula is described as follows^33^:

3 $$I=\frac{e}{U}\iint_{S} {2\pi ~r({\mu _e}{N_e}+{\mu _p}{N_p}+{\mu _n}{N_n})E \cdot }{E_L}drdz$$

where *U* represents the applied voltage, *S* indicates the cross-sectional area of discharge channel, and *E*_*L*_ signifies the Laplace electric field intensity within the discharge channel.

**Fig. 4:**
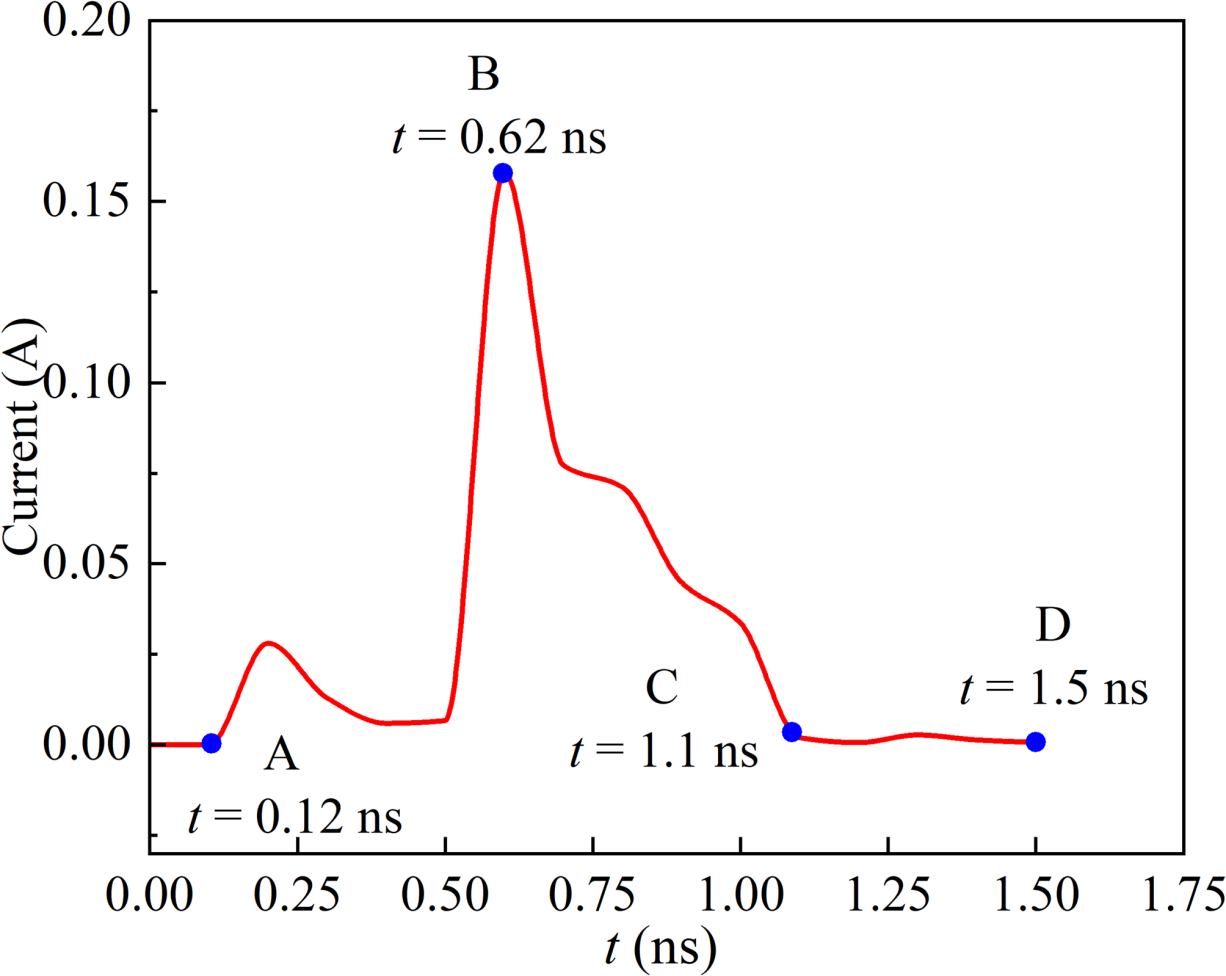
Space current pulse under reference condition.

As illustrated in Fig. 4, the space current pulse initiates an increase at *t* = 0.12 ns, reaches its peak at *t* = 0.62 ns, and stabilizes at *t* = 1.5 ns. Consequently, the pulse can be divided into three distinct stages: current rise (A-B), current fall (B-C), and current stabilization (C-D). Our simulation results show a high degree of consistency with the space current pulses reported in [17, 34], both in terms of the amplitude and time scale.

**Fig. 5:**
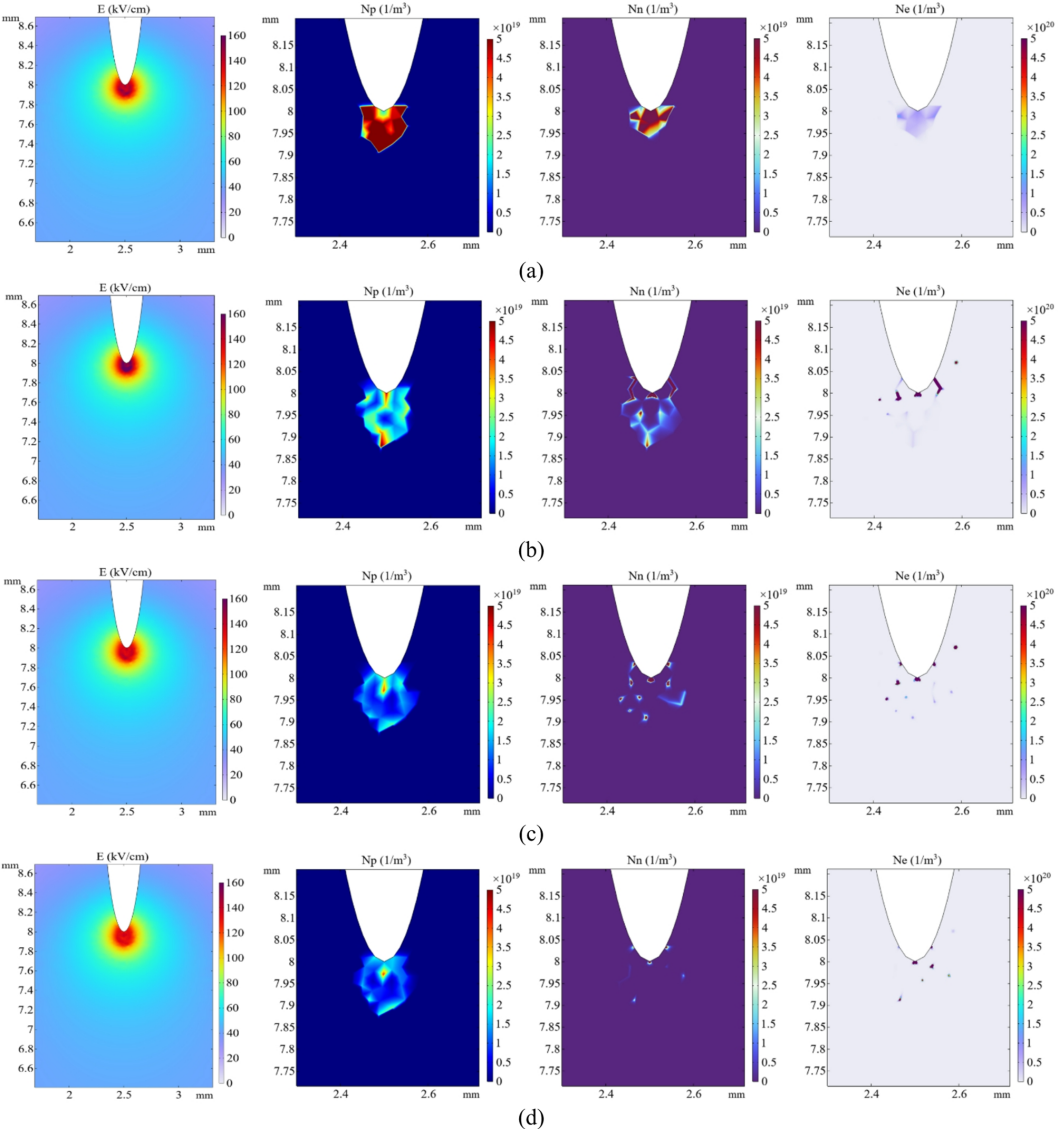
Distribution of electric field and charged particles. (**a**) 0.12 ns. (**b**) 0.62 ns. (**c**) 1.1 ns. (**d**) 1.5 ns.

Figure 5 depicts the electric field intensity as well as the number densities of positive ions, negative ions, and electrons at locations A, B, C, and D. To more clearly present the spatial distribution characteristics of charged particles in the discharge process, a smaller observation area is defined for their density distribution in comparison to the electric field distribution. In the first stage (see Fig. 5a, b), at the initial moment of discharge, the electric field distortion is small, and a weak ionization reaction occurs near the tip. The number of electrons and positive ions begins to increase, causing a minor peak in the current. However, as electrons migrate to the needle electrode, the total number of charged ions decreases, leading to a gradual decline in the current. Meanwhile, the continuous accumulation of positive ions strengthens the electric field near the tip, causing ionization reactions to gradually intensify and the migration speed of charged reaction s to increase. As a result, the current amplitude rapidly increases until it reaches a peak of 0.165 A. In the second stage (see Fig. 5b, c), certain positive ions interact with negative ions to form neutral molecules, leading to a gradual decrease in the space charge density and electric field strength; consequently, the current pulse experiences a sharp decline. In the third stage (see Fig. 5c, d), as the number of electrons near the tip continues to decrease and the reactions between ions are gradually weakened, the number of charged particles tends toward dynamic equilibrium, causing the current to stabilize.

## Influencing factors of space current pulse

### Influence of applied voltage

**Fig. 6:**
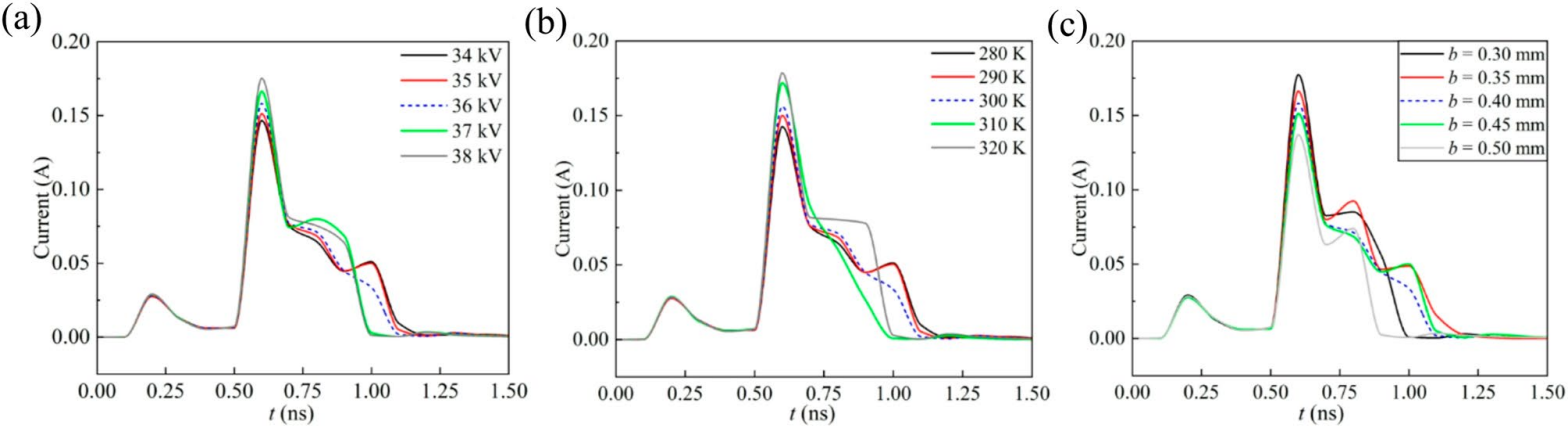
Current pulses at varying conditions. (**a**) applied voltages. (**b**) temperatures. (**c**) tip curvatures.

Assuming that the temperature (*T* = 300 K) and tip curvature (*a* = 2 mm and *b* = 0.4 mm) are maintained constant and then the applied voltage incrementally rises from 34 to 38 kV, the obtained space current pulses are depicted in Fig. 6a. With the enhancement in voltage, the amplitude of current pulse rises. This is attributed to the fact that, in the first stage, the enhanced electric field near the tip promotes ionization reactions, increases the number densities of positive ions and electrons, and accelerates the positive ions away from the needle electrode; in the second stage, the electrons within the space migrate toward the needle electrode more rapidly, the neutralization reactions between electrons and positive ions becomes faster, and the charged particles dissipate more quickly.

### Influence of temperature

Provided that the voltage (*V* = 36 kV) and tip curvature are kept the same and then the temperature gradually rises from 280 to 320 K, the results are presented in Fig. 6b. As the temperature rises, the peak value of current pulse increases. This is because in the first stage, the heightened collision frequency and collision energy between particles intensify ionization reactions near the tip and promote the formation of electron avalanches; in the second stage, the neutralization reactions between positive and negative ions, as well as between positive ions and electrons, are accelerated, resulting in a rapid decrease in space charge density.

### Influence of tip curvature

When the minor axis expands from 0.3 mm to 0.5 mm (notably, the variation in tip curvature is achieved through altering the minor axis), the space current pulses are shown in Fig. 6c. As the curvature increases (i.e., the minor axis b decreases), the peak value of current rises. This is attributed to the fact that, in the first stage, the distortion of electric field in the tip region is enhanced, leading to higher densities of electrons and positive ions; in the second stage, the migration of electrons toward the needle electrode is accelerated, the speed of attachment reactions between electrons and neutral particles are improved, and the neutralization reactions between electrons and positive ions are intensified.

## EM wave signals

### EM waves excited by current pulse in GIS

**Fig. 7:**
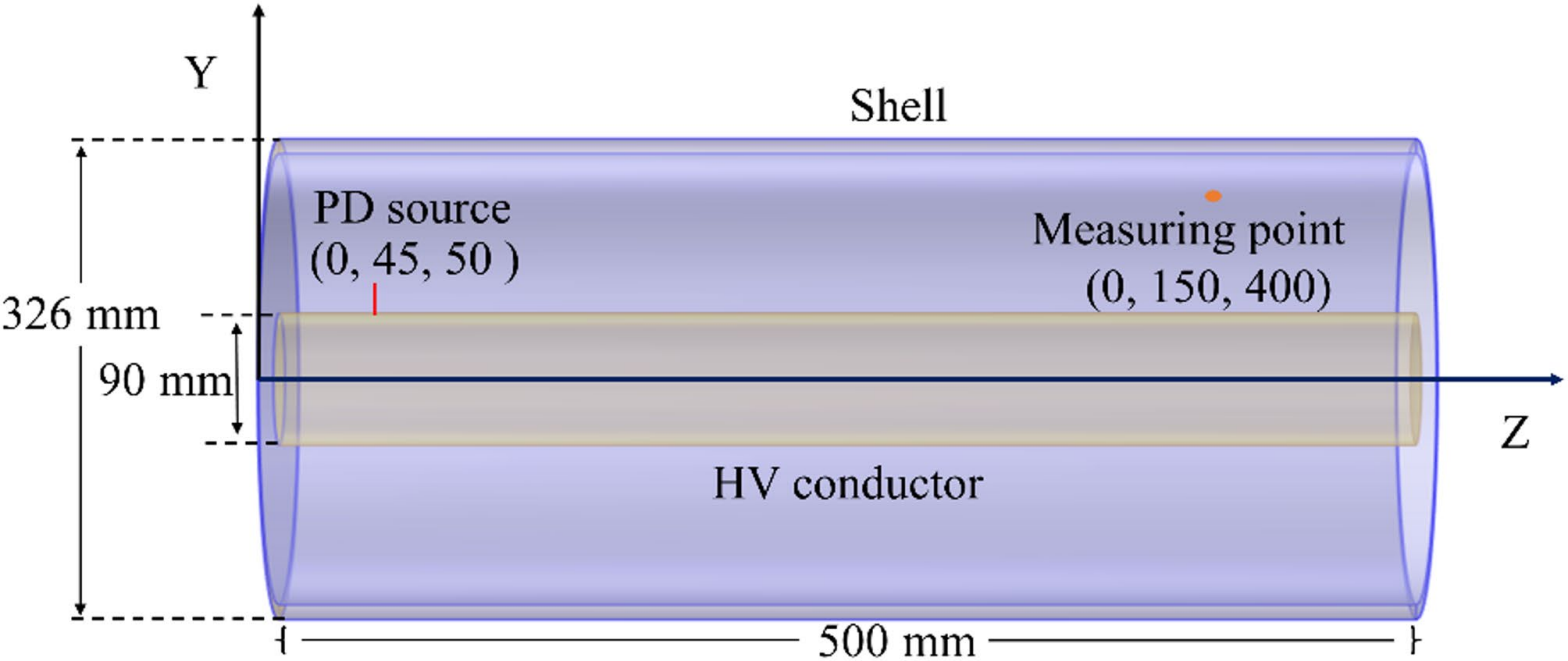
Simulation model of GIS.

This paper employs the FDTD method to discretize Maxwell’s equations, which can describe the generation and propagation of EM waves. Considering the stability of solutions in the time domain, the time step must comply with the following formula^35^:

4 $$c{\text{\varvec{\Delta}}}t \leqslant {(\frac{1}{{{\text{\varvec{\Delta}}}{x^2}}}+\frac{1}{{{\text{\varvec{\Delta}}}{y^2}}}+\frac{1}{{{\text{\varvec{\Delta}}}{z^2}}})^{ - \frac{1}{2}}}$$

where *c* represents the speed of light in vacuum, and Δ*x*, Δ*y*, and Δ*z* means the cell sizes. The simulation model utilizes a 36 kV straight GIS^36^. Its enclosure is constructed from aluminum alloy with a radius of 163 mm, the inner HV conductor is composed of copper with a radius of 45 mm, and the overall length is 500 mm. The PD source is located on the HV conductor and 50 mm from the left port, with its length corresponding to that of the discharge gap, as shown in Fig. 7. Considering that the PD detection sensor in practice is installed near the shell^37^, a probe is set to the coordinate (0, 150 mm, 400 mm) in the simulation.

### Simulation results

This section explores the influences of discharge stages and conditions on the amplitude-frequency characteristics of EM waves in the UHF range.

#### EM waves in time domain and their spectrum

**Fig. 8:**
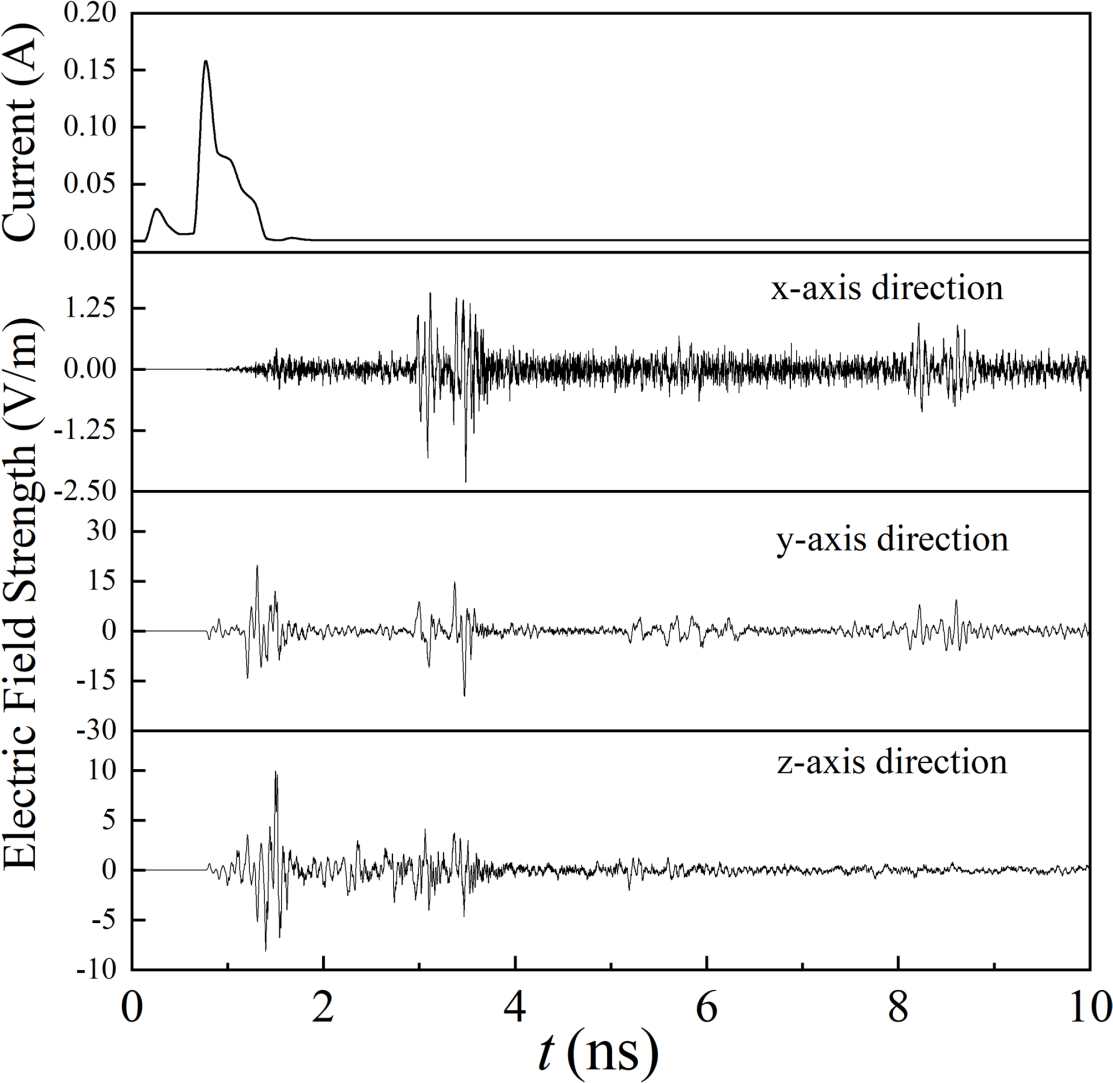
Time-domain waveforms.

In this section, the entire current pulse, produced under the reference condition, serves as the PD source. Figure 8 illustrates the time-domain waveforms, which are measured by the probe, along the x-, y-, and z-axes.

The EM wave signals exhibit a delay relative to the current pulse due to a certain distance between the measuring point and the PD source. In the initial discharge phase, the fluctuations in the current pulse are small, leading to the detection of only low-amplitude EM waves. When the current pulse reaches its peak value, a corresponding peak is observed in the EM wave signal. Subsequently, the signal undergoes continuous reflection and attenuation.

**Fig. 9:**
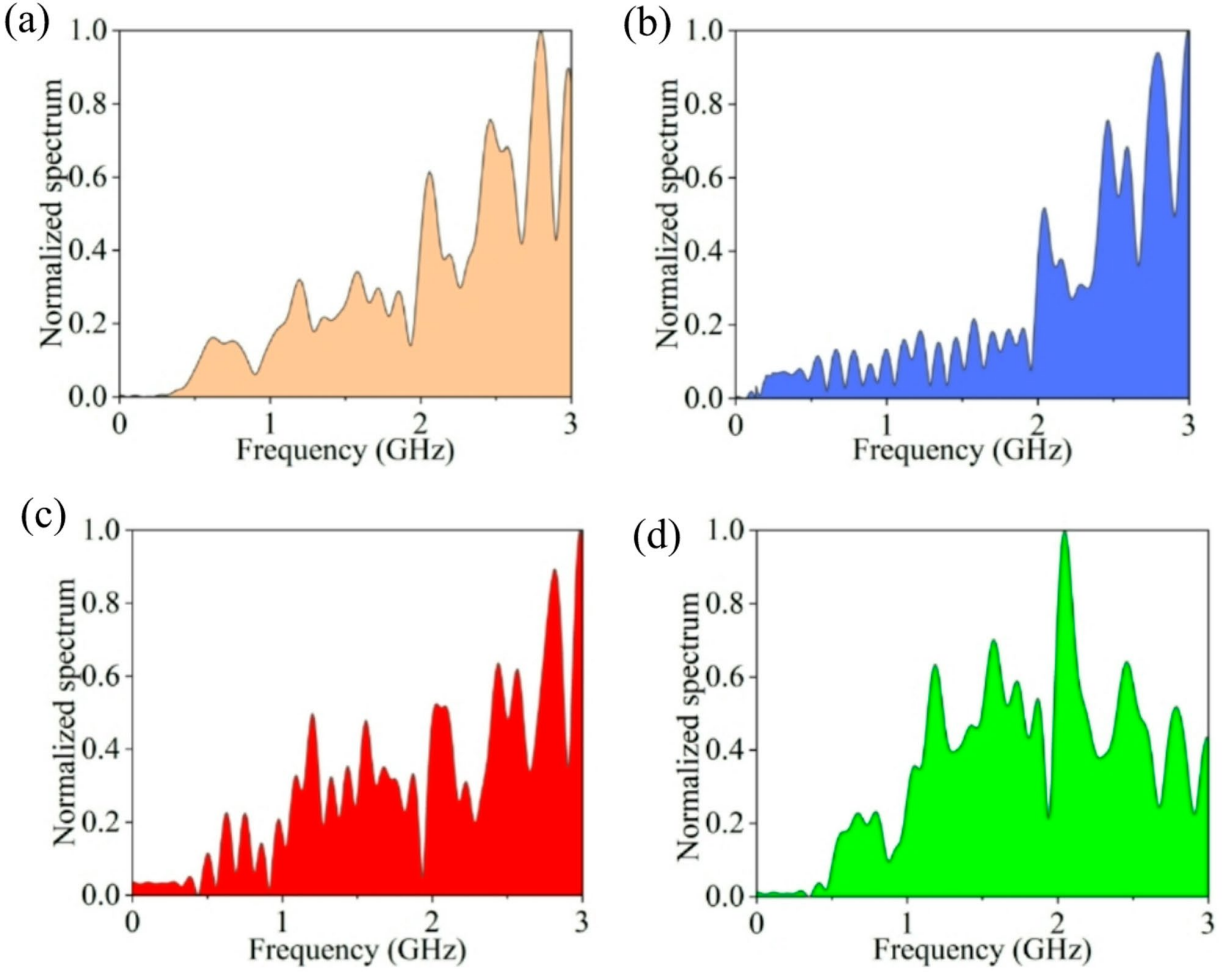
Spectrogram of EM waves corresponding to (**a**) complete signal. (**b**) rising stage. (**c**) falling stage. (**d**) stabilization stage.

The normalized spectrum corresponding to the resultant vector of the three components is shown in Fig. 9a. It is evident that the simulated signal exhibits relatively low strength within the 0–1 GHz range, whereas the signal strength increases significantly in the 1–3 GHz range. These phenomena closely resemble the EM wave characteristics measured experimentally using ultra-high-frequency (UHF) antennas, as reported in^38^, although there are slight differences. This is attributed to the idealized simulation model, as well as the incomplete consideration of certain physical phenomena, such as noise and measurement errors, in the simulation. Overall, the computational results show a good agreement with the experimental findings, thereby demonstrating the effectiveness and correctness of computed discharge current.

#### Spectrum distribution in different discharge stages

In this section, the time-domain waveforms corresponding to the three stages of the current pulse under the reference condition are utilized as PD sources, with the corresponding spectra presented in Fig. 9b, c, d. To investigate the amplitude-frequency characteristics of EM waves, we calculate the energy proportion of the frequency band in different discharge stages. The calculation procedure is described as follows. First, the total energy in each discharge stage is calculated according to the spectrum distribution. Then, the target frequency is divided into four bands^39^ namely, the low frequency band (0.2–0.9 GHz), mid-low frequency band (0.9–1.6 GHz), mid-high frequency band (1.6–2.3 GHz), and high frequency band (2.3–3.0 GHz), and the total energy in each frequency band is computed, respectively. Finally, the energy proportions of each frequency band across the different discharge stages are determined by dividing the energy of each frequency band by the total energy.

**Fig. 10:**
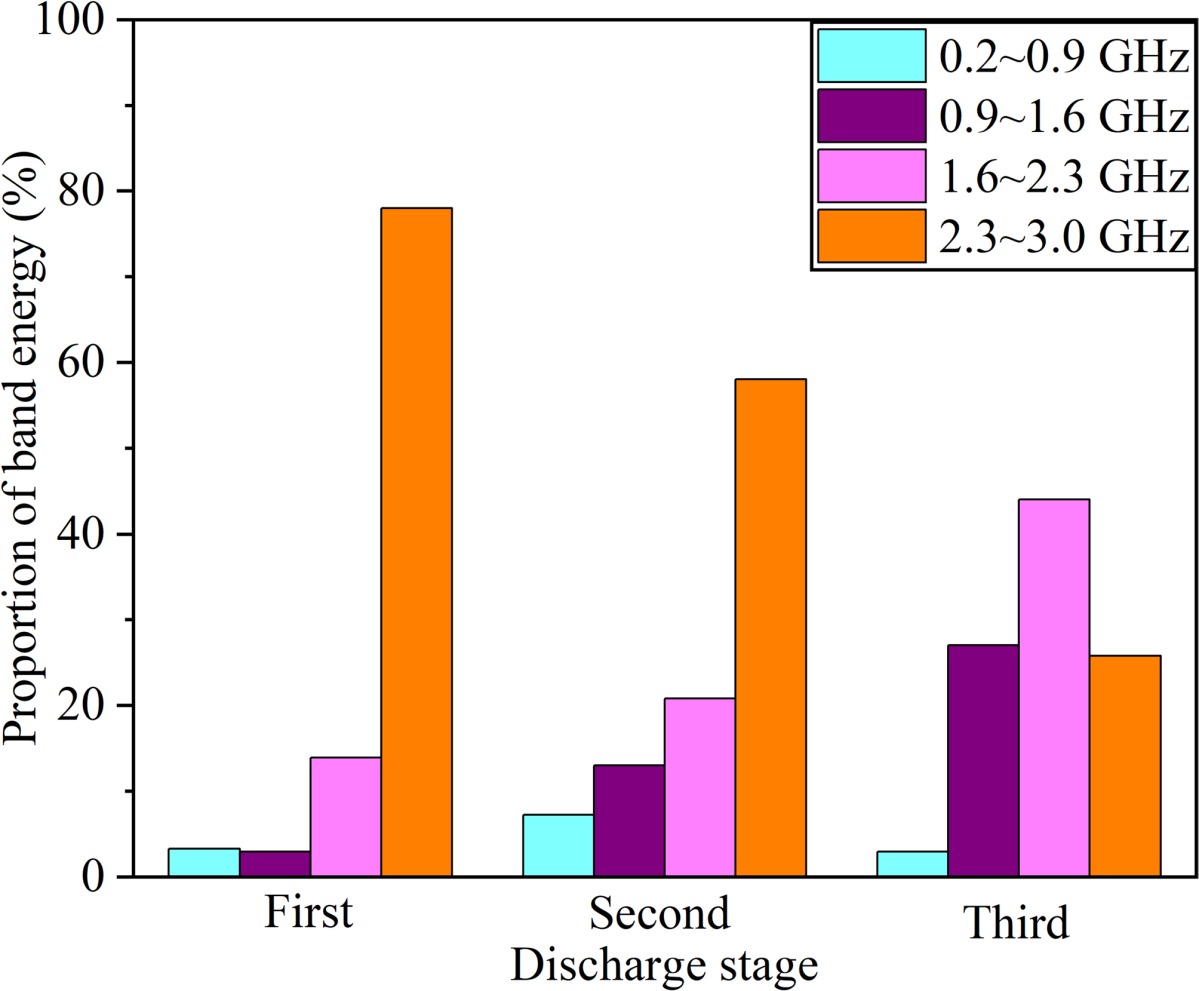
Energy Proportion in different discharge stages.

Figure 10 illustrates the spectral energy proportions in the three discharge stages. It can be seen that the spectral energy is predominantly concentrated in the high frequency band within both the rising and falling stages; however, compared to the former stage, there is a reduction in the ratio of energy in the high frequency band, and an increase is observed in other three frequency bands in the latter stage. This is attributed to the rapid formation of electron avalanches within the discharge channel, which leads to a swift rise in the space charge density in the rising stage; moreover, the continuous neutralization of charged particles results in a rapid reduction in the space charge density in the falling stage. Consequently, the rapid current pulses generated in this process can excite more high-frequency EM waves. In the stabilization stage, energy is uniformly distributed across three frequency bands: mid-low, mid-high, and high frequencies, with the highest concentration observed in the mid-high frequency band. This phenomenon can be attributed to the dynamic balance of space charge density. Furthermore, it is evident that the energy distribution across the three discharge stages remains minimal in the low-frequency band.

#### Energy proportion under different discharge conditions

**Fig. 11:**
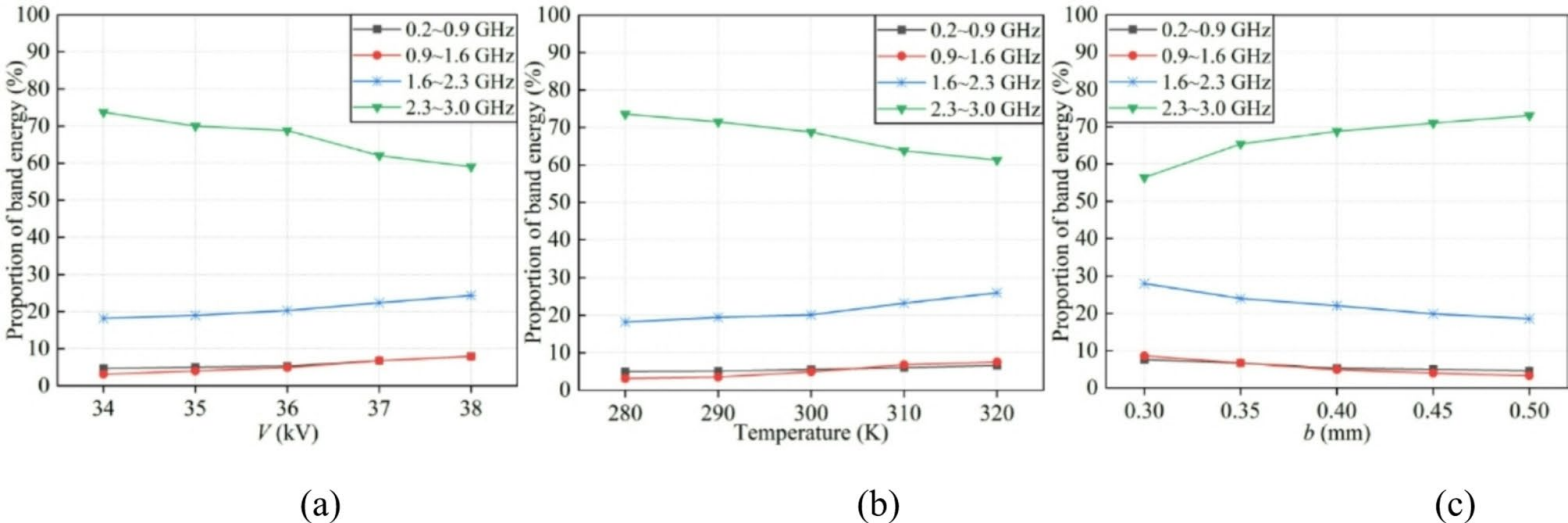
Energy proportion under various conditions. (**a**) Applied voltages. (**b**) Temperatures. (**c**) Tip curvatures.

Figure 11 depicts the spectral energy ratios under various discharge conditions. As the applied voltage elevates from 34 to 38 kV, the energy proportion in the high-frequency band decreases from 73.7 to 59.0%, whereas the energy proportions in the low, mid-low, and mid-high frequency bands increase by 3.2%, 4.8%, and 6.7%, respectively. When the temperature is elevated from 280 to 320 K, the energy proportion in the high-frequency band declines from 73.5 to 61.3%, accompanied by corresponding increases of 1.6%, 4.0%, and 7.6% in the low, mid-low, and mid-high frequency bands, respectively. Furthermore, as the minor axis length extends from 0.3 to 0.5 mm, the energy ratio for the high-frequency band rises from 56.4 to 73%, but those for the low, mid-low, and mid-high frequency bands decrease by approximately 3%, 5.1%, and 8.5%, respectively. It is evident that the rise of these factors, including *V*, *T*, and tip curvature, leads to a decreasing trend in the energy proportion within the high-frequency band whereas the energy proportions in other three frequency bands exhibit an upward trend. This can be attributed to the reduction in the duration of rapidly changing space currents and the increase in the duration of stable space currents. Specifically, the elevated voltage can accelerate electron motion, thereby enhancing the ionization reaction and facilitating the participation of a greater number of electrons in the discharge; besides, the increase in temperature can rise the collision frequency of gas molecules, therefore intensifying the ionization reaction and generating more charged particles; in addition, electrodes with a greater tip curvature can enhance the local electric field strength, thus accelerating the discharge process and producing a larger quantity of electrons.

## Conclusion

- The space current pulse amplitude is positively correlated with voltage, temperature, and tip curvature. This is attributed to the fact that the increase in these variables strengthens the electric field near the tip, accelerates ionization reactions, improves the migration rate of charged particles, and enhances the attachment reactions between electrons and neutral particles.
- In the rising and falling stages of current pulse, the space charge density presents rapid fluctuations, leading to a concentration of spectral energy in the high frequency band of the UHF range. Conversely, in the stabilization stage, the space charge density approaches dynamic equilibrium, resulting in a more uniform distribution of spectral energy in both the middle and high frequency bands.
- With the increase in applied voltage, temperature, or tip curvature, the proportion of energy in EM waves generated by positive corona discharge in SF_6_ shows an upward trend in the low, mid-low, and mid-high frequency bands, whereas the energy proportion within the high frequency band conforms to a downward trend.
- In our future work, we plan to establish a detection platform for EM waves excited by PD to indirectly validate the accuracy of computed discharge current by comparing experimental results with those from simulations.

## Data Availability

Data is provided within the manuscript or supplementary information files.
